# Augmented Twin-Nonlinear Two-Box Behavioral Models for Multicarrier LTE Power Amplifiers

**DOI:** 10.1155/2014/762534

**Published:** 2014-01-29

**Authors:** Oualid Hammi

**Affiliations:** Electrical Engineering Department, King Fahd University of Petroleum and Minerals, Dhahran 31261, Saudi Arabia

## Abstract

A novel class of behavioral models is proposed for LTE-driven Doherty power amplifiers with strong memory effects. The proposed models, labeled augmented twin-nonlinear two-box models, are built by cascading a highly nonlinear memoryless function with a mildly nonlinear memory polynomial with cross terms. Experimental validation on gallium nitride based Doherty power amplifiers illustrates the accuracy enhancement and complexity reduction achieved by the proposed models. When strong memory effects are observed, the augmented twin-nonlinear two-box models can improve the normalized mean square error by up to 3 dB for the same number of coefficients when compared to state-of-the-art twin-nonlinear two-box models. Furthermore, the augmented twin-nonlinear two-box models lead to the same performance as previously reported twin-nonlinear two-box models while requiring up to 80% less coefficients.

## 1. Introduction

Energy efficient wireless communication systems are being sought as part of the global concern for greener communication systems. Most of the energy savings can be made at the network level as well as the base station side. In this context, improving the efficiency of the radio frequency (RF) power amplifier (PA) is perceived as a highly attractive alternative that can enable higher efficiency transmitters and greener communication systems. However, achieving high efficiency amplification comes at the expense of severely nonlinear behavior due to the inherent efficiency-linearity dilemma in power amplifiers. Since linearity is a must, power efficient amplification circuits are always used along with a linearization technique which allows for mitigating nonlinear distortions of power amplifiers operating in their power efficient nonlinear region [1–3]. For modern base station applications, baseband digital predistortion technique is the preferred linearization method as it allows for acceptable linearity levels with a continuously increasing modulation bandwidth capability. One major advantage of baseband digital predistortion is its high flexibility and reconfigurability due to the digital implementation of the predistortion function and the availability of a wide range of functions that can compensate for static and dynamic distortions.

With the large adoption of baseband digital predistortion based linearizers, behavioral modeling of RF power amplifiers has received an increasing interest mainly motivated by the need to accurately predict the nonlinear behavior of the power amplifier for system level simulations, and especially the fact that the predistortion can be perceived as a reverse behavioral modeling problem [1]. A large variety of single-box and two-box structures have been reported for the modeling and predistortion of RF power amplifiers. Single-box models range from the comprehensive and computationally heavy Volterra series [4, 5] to the compact memory polynomial model [6] and its variants such as the envelope memory polynomial [7], the orthogonal memory polynomial [8], the hybrid memory polynomial-envelope memory polynomial [9], and the generalized memory polynomial model [10]. Single-box models often result in a large number of coefficients when used for highly nonlinear RF power amplifiers driven by multicarrier wideband signals. In such cases, two-box models appear as a valuable alternative to maintain the modeling performance while requiring a lower number of model coefficients. Popular two-box structures include the Wiener, Hammerstein and their augmented versions [11–13], and the twin-nonlinear two-box models [14].

In modern applications, gallium nitride (GaN) based Doherty power amplifiers are used along with multicarrier wideband long-term evolution (LTE) signals. GaN transistors offer superior performances compared to their laterally diffused metal oxide semiconductor (LDMOS) counterparts; however, they typically result in stronger memory effects [15]. These effects get even stronger when advanced amplifier circuits such as Doherty amplifiers with harmonically tuned carrier and peaking amplifiers are used. Such combination of amplification circuits (GaN based high efficiency amplifiers) and operating conditions (multicarrier LTE drive signals) require the development of advanced behavioral models. These models are expected to either outperform state-of-the-art existing models while requiring comparable number of coefficients or achieve similar performances as their state-of-the-art counterparts while requiring a lower number of coefficients.

In this paper, augmented twin-nonlinear two-box (ATNTB) models are reported for the behavioral modeling of GaN based Doherty power amplifiers driven by multicarrier wideband LTE signals. In order to accurately model the highly nonlinear behavior of the DUT which includes strong memory effects, the proposed models use a cascaded combination of a memory polynomial function with cross terms and a memoryless look-up table (LUT). The ATNTB models are experimentally validated and their performances are benchmarked against those of conventional twin-nonlinear two-box models. Experimental results using two power amplifiers prototypes clearly illustrate the superiority of the proposed models as they can achieve, for the same total number of coefficients, better performance than the previously reported ones. Furthermore, compared to the previously reported twin-nonlinear two-box models, the proposed models require a lower number of coefficients for the same performance.

In Section 2, the augmented forward and reverse twin-nonlinear two-box models are introduced. In Section 3, the devices under test used in this work as well as their AM/AM and AM/PM characteristics are described. In Section 4, the performance of the proposed ATNTB models is reported and benchmarked against that of the conventional twin-nonlinear two-box. The conclusion is summarized in Section 5.

## 2. Proposed Augmented Twin-Nonlinear Two-Box Models

To enable accurate modeling of highly nonlinear static and dynamic distortions generated by high efficiency power amplification circuits driven by wideband signals, the proposed models use a combination of a strongly nonlinear static distortions function and a mildly nonlinear dynamic distortions function. The static nonlinear function can be implemented using a look-up table or a memoryless polynomial function. The nonlinear dynamic distortions are modeled by a memory polynomial function with cross terms. The separation of the static and dynamic distortions allows for the use of a reduced nonlinearity order in the memory polynomial function which will result in a lower number of coefficients than single-box models as it was previously reported for the case of the twin-nonlinear two-box models [14]. The use of the cross-terms improves the capability of the memory polynomial function in mimicking the dynamic behavior of the device under test.

**Figure 1:**
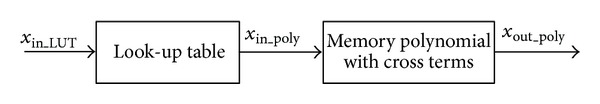
Block diagram of the proposed augmented forward twin-nonlinear two-box model.

**Figure 2:**
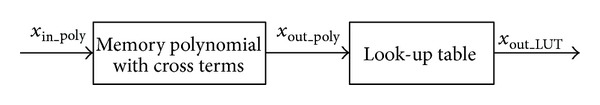
Block diagram of the proposed augmented reverse twin-nonlinear two-box model.

In this paper, the augmented twin-nonlinear two-box models are obtained by connecting the memoryless nonlinear function, implemented as a look-up table, and the dynamic nonlinear function, implemented using a memory polynomial function with cross terms, in a cascaded fashion. The augmented forward twin-nonlinear two-box model is obtained when the LUT precedes the memory polynomial function as illustrated in Figure 1. Conversely, the augmented reverse twin-nonlinear two-box model is obtained when the memory polynomial function is connected upstream of the LUT as shown in Figure 2.

In the proposed models, the memory polynomial function with cross terms is implemented according to

(1) xout_Poly(n) =∑j=0M1∑i=1N1aij·xin_Poly(n−j)·|xin_Poly(n−j)|i−1  +∑j=0M2∑i=2N2∑l=1L2bijl·xin_Poly(n−j)·|xin_Poly(n−j−l)|i−1  +∑j=0M3∑i=2N3∑l=1L3cijl·xin_Poly(n−j)·|xin_Poly(n−j+l)|i−1,

where  *x*_in_Poly_  and  *x*_out_Poly_  are the input and output baseband waveforms of the memory polynomial function, respectively.  *N*_1_,  *N*_2_, and  *N*_3_  are the nonlinearity orders of the aligned, lagging, and leading terms, respectively.  *M*_1_,  *M*_2_, and  *M*_3_  represent the memory depths of the aligned, lagging, and leading polynomial functions, respectively.  *L*_2_, and  *L*_3_  represent the lagging and leading cross terms orders, respectively.  *a*_*ij*_,  *b*_*ijl*_, and  *c*_*ijl*_  are the coefficients of the aligned, lagging, and leading polynomial functions, respectively.

In the proposed models, the nonlinearity orders as well as the memory depths of the aligned, lagging, and leading polynomial functions were set to equal values. Similarly, the number of leading and lagging cross terms used was equal:

(2) $$\begin{array}{c} N_{1}=N_{2}=N_{3}=N,\\ M_{1}=M_{2}=M_{3}=M,\\ L_{2}=L_{3}=L. \end{array}$$

However, if needed, the nonlinearity orders and memory depths of the polynomial functions as well as the number of leading and lagging cross terms can be optimized independently.

In the conventional twin-nonlinear two-box model, the memory polynomial function is given by the first term of (1); that is,

(3) $$\begin{array}{c} x_{\text{out}\_\text{Poly}}\left(n\right)=\sum_{j=0}^{M_{1}}\sum_{i=1}^{N_{1}}a_{ij}\cdot x_{\text{in}\_\text{Poly}}\left(n-j\right)\cdot {\left|x_{\text{in}\_\text{Poly}}\left(n-j\right)\right|}^{i-1}, \end{array}$$

where all the variables are the same as those defined in (1).

## 3. Device under Test Characterization

The ATNTB models were applied to model two GaN based Doherty power amplifiers. The first Doherty amplifier operates around 2425 MHz [16]. This device under test (DUT) was tested using a 4-carrier LTE signal with a carrier configuration of 1001 (where 1 refers to the ON carriers and 0 refers to the OFF carriers) and a total bandwidth of 20 MHz. The second device is also a GaN based Doherty amplifier [17]. This DUT operates around 2140 MHz and was tested using a single carrier LTE signal having a bandwidth of 20 MHz.

**Figure 3:**
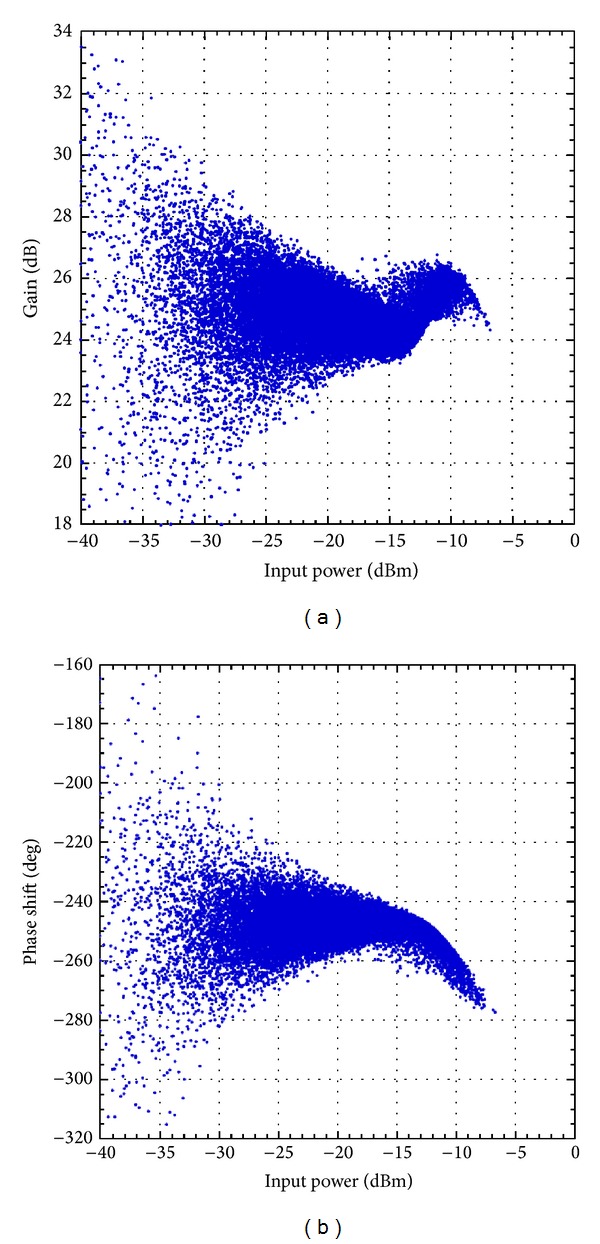
Measured AM/AM and AM/PM characteristic of the 2425 MHz Doherty PA driven by the 4-carrier LTE signal. (a) AM/AM characteristic and (b) AM/PM characteristic.

**Figure 4:**
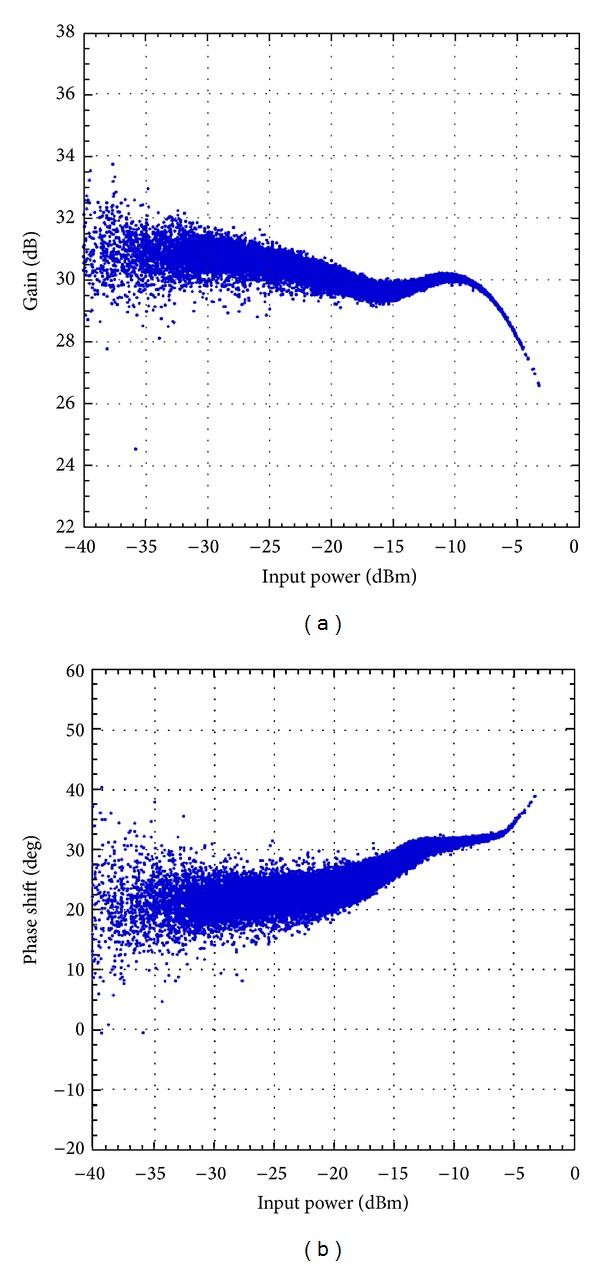
Measured AM/AM and AM/PM characteristic of the 2140 MHz Doherty PA driven by the 4-carrier LTE signal. (a) AM/AM characteristic and (b) AM/PM characteristic.

First, the Doherty amplifiers were characterized by acquiring their input and output baseband complex waveforms. The technique thoroughly described in [1] consists in using the baseband complex waveform measured at the output of the DUT with a vector signal analyzer along with its counterpart at the input of the DUT. The measurements were processed to time align the measured waveforms and then derive the AM/AM and AM/PM characteristics of each amplifier. These characteristics are reported in Figures 3 and 4 for the 2425 MHz Doherty amplifier and the 2140 MHz Doherty amplifier, respectively.

According to the AM/AM and AM/PM characteristics, both amplifiers have a strongly nonlinear behavior mainly due to the Doherty configuration. Comparing the curves reported in Figures 3 and 4 shows that a more pronounced dispersion is observed in the case of the 2425 MHz Doherty PA. This can be attributed partly to the nature of the signal with noncontiguous carriers. Indeed, it was shown that, for the same total bandwidth, signals with noncontiguous carriers emulate stronger memory effects than signals with contiguous carriers [18]. To quantitatively evaluate the strength of the memory effects of each DUT, the memory effects intensity metric was computed using the memoryless postcompensation technique reported in [18]. The memory effects intensity was calculated using the entire observation bandwidth. The results reported in Table 1 clearly illustrate the importance of the memory effects in the first device under test. Conversely, the memory effects exhibited by the second device under test (the 2140 MHz Doherty PA) are moderate. Accordingly, it is expected that the proposed models which enhances the memory effects modeling capabilities will result in more significant improvement when applied to model the 2425 MHz Doherty PA.

## 4. Performance Assessment of Augmented Twin-Nonlinear Two-Box Models 

The augmented forward and reverse twin-nonlinear two-box models were identified for each of the devices under test. For conciseness, thorough results will be provided for the 2425 MHz Doherty PA; then, a summary of the results obtained for the second DUT will be reported. In order to compare the performances of the augmented forward twin-nonlinear two-box (AFTNTB) with those of the forward twin-nonlinear two-box (FTNTB) and evaluate the improvement achieved by including the cross terms, the LUT subfunction of the FTNTB model was built. Then, the data were de-embedded to the memory polynomial function's input and output planes. The same data were used to identify the memory polynomial function of the FTNTB model and the memory polynomial function with cross terms of the augmented FTNTB model.

**Figure 5:**
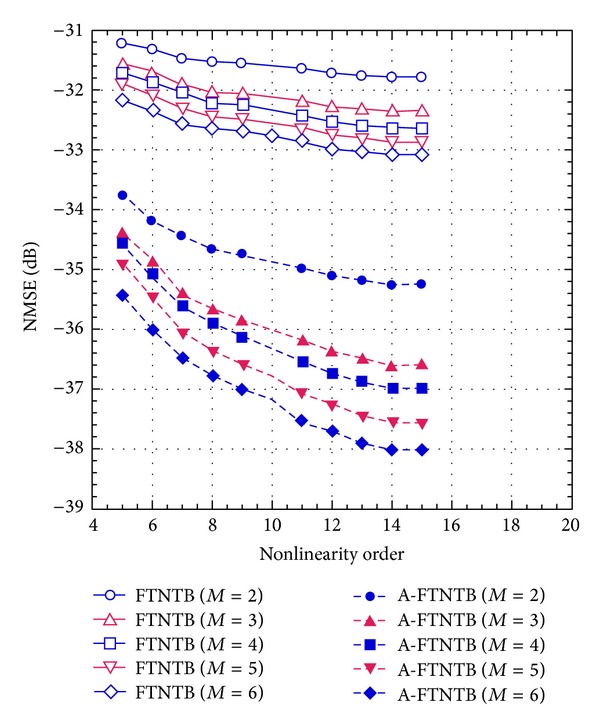
NMSE performance of the forward twin-nonlinear two-box model and the augmented forward twin-nonlinear two-box model as function of the nonlinearity order.

First, the FTNTB model was identified using the measured data for a wide set of nonlinearity orders and memory depths. The nonlinearity order (*N*_1_) was varied from 5 to 15 and the memory depth (*M*_1_) was swept from 2 to 10. For each set of coefficients, the performance of the FTNTB model was evaluated in terms of the normalized mean-square error (NMSE). Then, the AFTNTB model was identified for the same ranges of the nonlinearity order and memory depth and for a leading and lagging cross terms order (*L*) of 1. For each set of coefficients, the performance of the AFTNTB model was evaluated in terms of its NMSE. The results are reported in Figure 5. For clarity reasons, these results include the NMSE values obtained for a memory depth up to 6. Higher values of memory depths were found to result in marginal NMSE improvement and are thus not included in the figure. Figure 5 demonstrates the performance enhancement obtained with the proposed model by including the first order cross terms. Indeed, a 3 dB NMSE improvement is obtained when the AFTNTB model is used. The performances of both the state-of-the-art model and the proposed one improve as the nonlinearity order and/or the memory depth of the model increases. However, the proposed ATNTB model consistently outperforms the conventional one by more than 3 dB when the same nonlinearity order and memory depth are used for both models.

It is important to recall that, according to (1) and (3), for a given nonlinearity order and memory depth, the AFTNTB model has more coefficients than the FTNTB model. In fact, the total number of coefficients in the FTNTB and its augmented counterpart are given by (4) and (5), respectively:

(4) $$\begin{array}{c} K_{\text{FTNTB}}=T+N_{1}\times \left(M_{1}+1\right), \end{array}$$

(5) KAFTNTB=T+N1×(M1+1)+(N2−1)×(M2+1)×L2+(N3−1)×(M3+1)×L3,

where  *K*_FTNTB_  and  *K*_AFTNTB_  are the total number of coefficients (including the LUT subfunction) of the FTNTB and the AFTNTB, respectively.  *T*  is the size of the look-up table subfunction.  *N*_1_,  *M*_1_,  *N*_2_,  *M*_2_,  *N*_3_,  *M*_3_,  *L*_2_, and  *L*_3_  are those defined for (1).

Since the LUT subfunction is the same for both models, and in order to compare the relative complexity of the FTNTB and the AFTNTB models, the total number of coefficients in the polynomial subfunction will be considered. Given that the structures of the two models are similar, their relative complexity is solely function of the number of coefficients in the memory polynomial function. For the conditions specified by (2), one can find that

(6) $$\begin{array}{c} P_{\text{FTNTB}}=N\times \left(M+1\right), \end{array}$$

(7) $$\begin{array}{c} P_{\text{AFTNTB}}=N\times \left(M+1\right)+\left(N-1\right)\times \left(M+1\right)\times 2L, \end{array}$$

where  *P*_FTNTB_  and  *P*_AFTNTB_  are the total number of coefficients in the memory polynomial function of the FTNTB and the AFTNTB, respectively.

**Figure 6:**
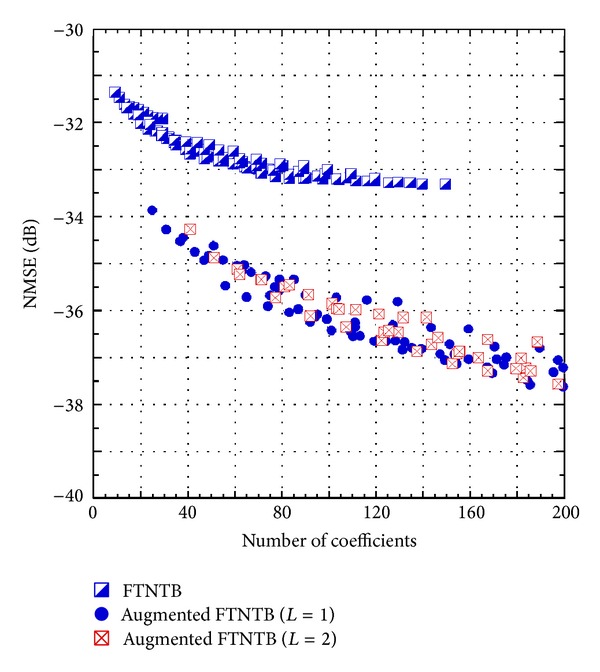
NMSE performance of the forward twin-nonlinear two-box model and the augmented forward twin-nonlinear two-box model as a function of the number of coefficients.

Equation (7) shows that, for the same nonlinearity order and memory depth, the total number of coefficients in the AFTNTB model is significantly higher than that of the FTNTB one. In order to get a better insight of the relative complexity of the two models, the NMSE is plotted as a function of the polynomial function's number of coefficients for the FTNTB model and the AFTNTB model. The results are summarized in Figure 6. Here, it is worth to mention that, in behavioral modeling, a better model is the one that can lead to the same performance as a benchmark model with less complexity, or better performance than a benchmark model for the same complexity. According to the results of Figure 6, one can note the superiority of the proposed model. Indeed, for the same number of coefficients, it achieves an NMSE improvement of approximately 2 to 3 dB. Furthermore, with 26 coefficients in the polynomial function, the FTNTB model leads to an NMSE of −31.7 dB while the AFTNTB model has an NMSE of −33.8 dB for the same number of coefficients. This NMSE (−33.8 dB) cannot be obtained with the conventional FTNTB model even when 150 coefficients are used in the polynomial function.

**Figure 7:**
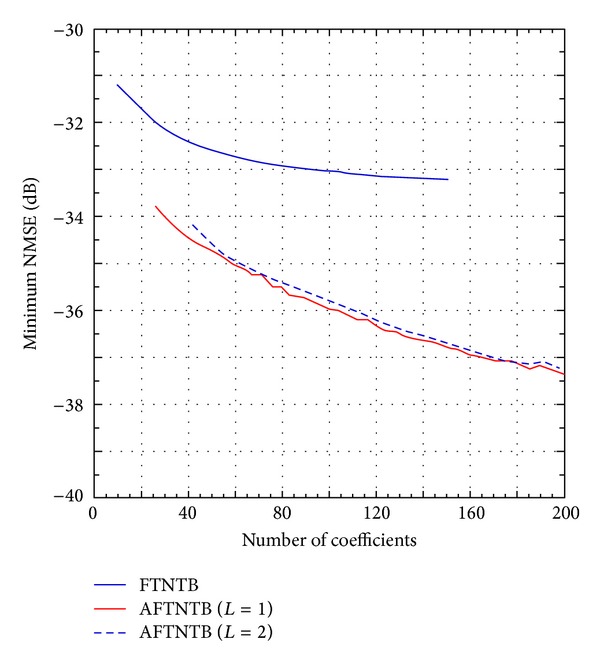
The best NMSE performance of the forward twin-nonlinear two-box model and the augmented forward twin-nonlinear two-box model as a function of the number of coefficients.

Figure 7 presents a summary of the results obtained for the FTNTB and the AFTNTB models when applied to predict the behavior of the 2425 MHz Doherty PA. This figure reports the minimum NMSE that can be obtained by each model as a function of the number of coefficients used in the memory polynomial function. This figure provides a clearer view of the results observed in Figure 6. Furthermore, it shows that increasing the cross terms index in the AFTNTB model from  *L* = 1  to  *L* = 2  does not lead to any improvement for the same number of coefficients. For higher number of coefficients, the AFTNTB model with  *L* = 2  can lead to an NSME as low as −39.1 dB but with 568 coefficients in the memory polynomial function. Since the main aim of the work is to propose a low complexity higher accuracy behavioral model, there is no need, for the considered DUT, to increase the cross terms index to  *L* = 2.

**Figure 8:**
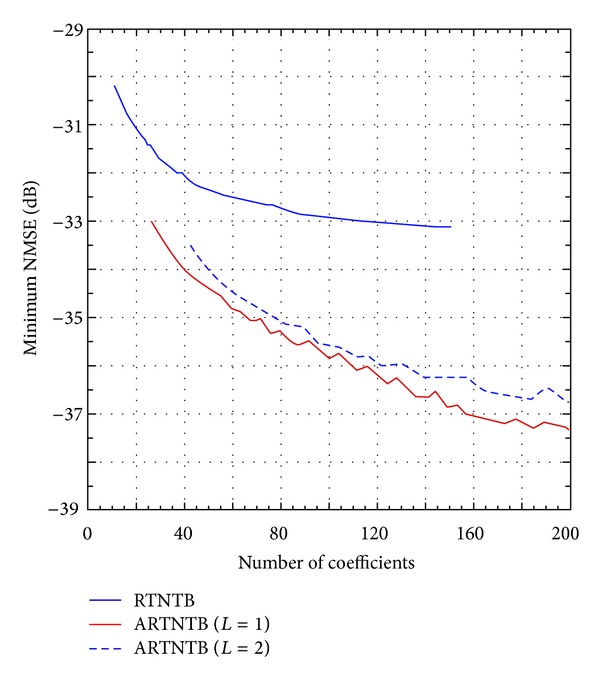
The best NMSE performance of the reverse twin-nonlinear two-box model and the augmented reverse twin-nonlinear two-box model as a function of the number of coefficients.

The results obtained when the reverse twin-nonlinear two-box (RTNTB) model and the augmented reverse twin-nonlinear two-box (ARTNTB) model are applied to model the 2425 MHz Doherty PA were also derived. The procedure used for the models identification and performance assessment is similar to the one described above for the FTNTB and the AFTNTB model.  Figure 8 reports the minimum NMSE obtained for the RTNTB and ARTNTB models as a function of the number of coefficients in the memory polynomial function. As demonstrated by the results reported in Figure 8, the ARTNTB model outperforms the RTNTB model with approximately 2 dB improvement in the NMSE for the same number of coefficients.

**Figure 9:**
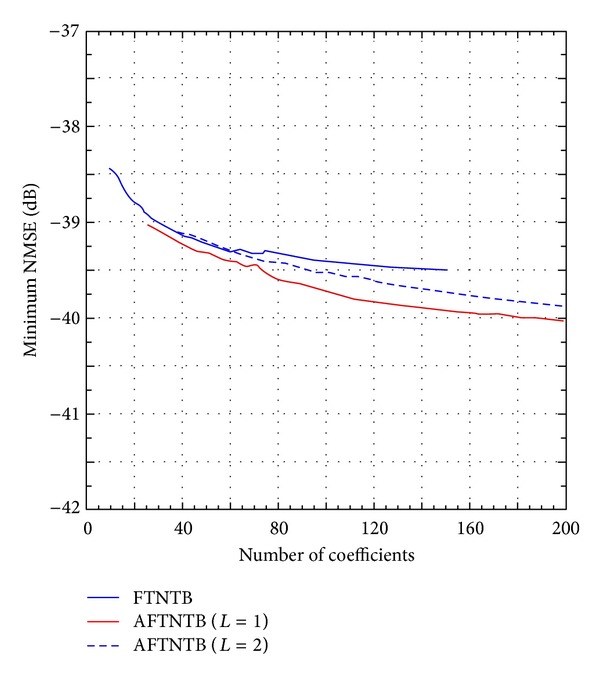
The best NMSE performance of the forward twin-nonlinear two-box model and the augmented forward twin-nonlinear two-box model for the 2140 MHz-Doherty PA.

The study described above was performed on the second device under test. For this 2140 MHz Doherty PA, the FTNTB model, AFTNTB model, RTNTB model, and ARTNTB models were derived for various nonlinearity orders and memory depths and their performances were compared.  Figure 9 presents the minimum NMSE obtained for the case of the FTNTB model and the AFTNTB model as a function of the number of coefficients in the memory polynomial subfunction. These results illustrate a minor improvement in the NMSE when the AFTNTB model is used. Similar results were obtained for the RTNTB and its augmented counterpart. These results are expected since the second device under test exhibits weaker memory effects than the 2425 MHz-Doherty PA as it can be concluded from the memory effects intensity calculated in the previous section. Under such conditions, the twin-nonlinear two-box models and their augmented versions perform similarly. Indeed, the main advantage of the augmented TNTB model is its ability to model strong memory effects for which the conventional TNTB models have limited capabilities.

## 5. Conclusion

In this paper, enhanced behavioral models suitable for high efficiency power amplifiers driven by broadband LTE signals are proposed. The labeled augmented forward and reverse twin-nonlinear two-box models are constructed using the cascade of a LUT and a memory polynomial function with cross terms. The experimental validation demonstrated the advantage of the proposed models in mimicking strong memory effects. Indeed, compared to their state-of-the-art counterparts, the proposed models combine two highly desirable features which are lower complexity and better accuracy. These models can be beneficially applied to power amplifiers behavioral modeling and predistortion in future LTE-advanced context.

## Figures and Tables

**Table 1:** Memory effects intensity of the devices under test.

Device under test ↓	Memory effects intensity	
	Lower channel	Upper channel
2425 MHz Doherty PA	17.49 dBc	21.23 dBc
2140 MHz Doherty PA	42.56 dBc	41.90 dBc
